# Pregnant women’s perspectives on integrating preventive oral health in prenatal care

**DOI:** 10.1186/s12884-021-03750-4

**Published:** 2021-04-01

**Authors:** A. Adeniyi, L. Donnelly, P. Janssen, C. Jevitt, B. Kardeh, H. von Bergmann, M. Brondani

**Affiliations:** 1 Department of Oral Health Sciences, Faculty of Dentistry, University of British Columbia, 2199 Wesbrook Mall, Vancouver, BC Canada; 2 Department of Oral Biological & Medical Sciences, Faculty of Dentistry, The University of British Columbia, Vancouver, Canada; 3 School of Population and Public Health, University of British Columbia, Vancouver, Canada; 4 Department of Family Practice, Faculty of Medicine, University of British Columbia, Vancouver, Canada; 5 Graduate Program in Rehabilitation Sciences, Faculty of Medicine, University of British Columbia, Vancouver, Canada

**Keywords:** Oral health, Prenatal care, Interprofessional education, Integrated care, Collaboration

## Abstract

**Background:**

Oral diseases are considered a silent epidemic including among pregnant women. Given the prevalence of oral conditions among pregnant women and the reported association with adverse pregnancy outcomes, there have been suggestions for the inclusion of preventive oral care in routine prenatal care. However, due to the different administrative and funding structure for oral health and prenatal care in Canada, progress towards this integration has been slow. Our study sought to qualitatively explore the views of pregnant women in British Columbia (BC) on the strategies for integrating preventive oral health care into prenatal care services.

**Methods:**

A qualitative approach was utilized involving semi-structured interviews with fourteen (14) purposefully selected pregnant women in Vancouver and Surrey, BC. The interviews were audio-recorded and transcribed. The transcripts were analyzed using an inductive thematic approach. Study validity was ensured via memoing, field-notes, and member checking.

**Results:**

Interviews ranged from 28 to 65 min producing over 140 pages of transcripts. Analysis resulted in three major themes: oral health experiences during pregnancy, perspectives on integration and integrated prenatal oral care, and strategies for addressing prenatal oral health care. A majority of participants were supportive of integrating preventive oral care in routine prenatal services, with referrals identified as a critical strategy. Oral health education was recognized as important before, during, and after pregnancy; oral health assessments should therefore be included in the prenatal care checklist. Limited funding was acknowledged as a barrier to oral health care access, which may explain why few participants visited their dentists during pregnancy. Interprofessional education surfaced as a bridge to provide prenatal oral health education.

**Conclusion:**

Pregnant women interviewed in this study support the inclusion of educational and preventive oral care during prenatal care, although their views differed on how such inclusion can be achieved in BC. They advocated the establishment of a referral system as an acceptable strategy for providing integrated prenatal oral health care.

**Supplementary Information:**

The online version contains supplementary material available at 10.1186/s12884-021-03750-4.

## Background

Oral diseases such as dental decay and periodontitis have been described as a silent epidemic affecting a large proportion of the population, and often remain unrecognised, undiagnosed and untreated [1]. During pregnancy, oral diseases have been associated with adverse pregnancy outcomes including gestational diabetes, preeclampsia, and the delivery of preterm and/or low birth weight babies [2]. Furthermore, poor maternal oral health is recognized as a risk factor for early childhood caries [3]. Therefore, preventive oral care including education is considered important during pregnancy and beyond [4].

Research findings indicate that providing preventive oral care during pregnancy reduces the incidence and severity of oral diseases [5]; thus it has a positive impact on women’s oral health, well-being, and quality of life [6]. Research evidence also suggests that many women do not seek or receive any oral care, including prevention and education, during pregnancy [7–10], despite exhibiting symptoms or signs of oral disease. Reasons for the low utilization of dental services include low oral health awareness and financial barriers [7, 9, 11]. Another reason may be that oral health is not routinely addressed during prenatal care, either because oral health providers are not usually members of the prenatal team or because there is little interprofessional education that includes oral health [12]. Integrating preventive oral health in routine prenatal services based on the concept of integrated care has, therefore been recommended [13].

The World Health Organization (WHO) describes integrated care as a polymorphous concept whose definition is shaped by the health system stakeholder’s perspective. To coalesce the ideas arising from the different views, they suggest that integrated care should be centered on the needs of individuals, their families, and the community [14]. A variety of approaches are available for achieving integration, from linkages to coordination in networks cooperation, and full integration [15]. Linkages happen when there is communication between professionals and adequate patient referrals between units, while coordination in networks involves sharing clinical information and managing the transition of patients between units [15]. For cooperation, network managers are engaged in improving contact between the units while for full integration, resources are pooled between different units. However from the perspective of oral health providers, researchers and funders, no single approach is considered the gold standard for integrating oral health into general health care settings, [16].

A pilot study concerning oral health during pregnancy explored the perspectives of socially disadvantaged women in British Columbia (BC) and highlighted their interest in an integrated approach for oral health care [8]. We, therefore, recognised the need to further explore pregnant women’s perspectives on potential strategies for integrating preventive oral health into routine prenatal care services. Our study is part of a larger inquiry exploring the views of stakeholders, including health care providers. The research question for this qualitative study was “What strategies do pregnant women in BC support for integrating preventive oral health into routine prenatal care services?”

## Methods

This exploratory qualitative study has a social constructivist orientation, which assumes that the research output is a co-construction between the researcher and study participants [17]. Data collection was based on semi-structured interviews. We used purposeful sampling to assure the attainment of critical representation of experiences and ideas by seeking maximum variation of the study participants. This study was approved by the University of British Columbia Behavioural Research Ethics Board (H18–01646) and all methods were conducted in accordance with relevant guidelines and regulations.

Participants included women at any stage of pregnancy, who were residents of Vancouver or Surrey, BC and were living in Canada for at least 1 year before the study commenced. Advertisements were placed at five prenatal care facilities in Vancouver and Surrey BC, and on social media platforms targeting local pregnant women (Instagram and private Facebook groups focused on prenatal discussions). Prospective participants were instructed to email the graduate student (AA), who then shared the study information sheet. Once participation was confirmed, written informed consent forms were sent via email and available dates and times for the interview were requested; all interviews took place via telephone to comply with physical distancing requirements due to the COVID-19 pandemic.

We conducted the interviews using a semi-structured interview guide (Additional file 1) containing open-ended questions developed from the literature. The questions were attached to a short vignette illustrating common oral health challenges during pregnancy. The vignette aimed at exploring the participants’ experiences and views on the delivery of preventive oral care during pregnancy, and the potential strategies for providing integrated prenatal oral health services in BC. We posed questions such as, “*Based on the vignette,* w*hat are your thoughts on the necessity of preventive oral care during pregnancy*? *What, in your opinion, is integration? What is your view on integration of oral health into prenatal care? What sort of preventive oral healthcare services do you think can be provided during pregnancy? How do you think your suggestion can be achieved?”.* In the last part of the interview, we explored their views on an existing model of integrated prenatal oral health [18]. The interviews were audio-recorded and transcribed verbatim. All participants completed a brief questionnaire to capture socio-demographic characteristics and help contextualize our findings. This information was employed for illustration purposes only; no identifiers were used throughout the study. Memoing, was performed against the field notes during the interviews, with the intention of increasing rigor and transparency. Data collection ended when no new information emerged [17].

Data analysis started after the first interview, using an inductive analytic approach while adopting a constant comparative method. Data analysis involved reading and re-reading the transcripts to foster understanding of the ideas and concepts discussed. Interview transcripts were coded using a thematic approach and *N-Vivo 12®* software*.* Primary codes were attached to excerpts to describe the essence of the narratives and to identify key ideas. Closely related codes were coalesced into subthemes, with major themes including several subthemes. Three of the authors (AA, MB, and CJ) developed the emerging codes, subthemes, and themes, by analyzing the transcript separately; they met to compare their work and ensure inter-coder consistency. Two of the authors (AA and KB) continued separate thematic analyses of the remaining transcripts separately and developed an audit trail to assess the accuracy of the study findings and interpretations. Memoing progressed during data analysis by recording emerging ideas to further enhance the audit trail for validation of study findings. Member checking was also performed to allow participants to correct errors, challenge interpretations of the transcript, and add insights.

## Results

We conducted a total of 14 semi-structured interviews with pregnant women in Vancouver and Surrey, BC, between March and July 2020, with repetition of ideas observed at the 9th interview. As shown in Table 1, pseudonyms were assigned to each participant and demographic information was summarized. Overall, the study participants were from diverse ethnic groups, their ages ranged from 25 to 40 years, and the majority had either a university or college level education. In addition, six women out of the 14 were primigravida.

**Table 1:** Socio-demographic characteristics of the study participants

No	Pseudonym	Age	Ethnicity	Highest Educational attainment	Employment status	History of pregnancy	Trimester of pregnancy
1	(PW23) Wendy	33	Caucasian	University Education	Full-time Employment	Multigravida (2nd Pregnancy)	Second
2	(OPW24) Xena	33	Caucasian	College Diploma	Full-time Employment	Primigravida	Third
3	PW26) Zuriel	40	South American	University Education	Part-time Employment	Multigravida (3rd Pregnancy)	First
4	(PW27) Anna	32	African/ Canadian	University Education	Full-time Employment	Multigravida (3rd Pregnancy)	Second
5	(PW28) Bailey	30	African	University Education	Unemployed	Multigravida (2nd Pregnancy)	Third
6	(PW29) Chloe	29	Pakistani	Postgraduate	Stay-at-home mom	Multigravida (3rd Pregnancy)	Third
7	(PW30) Daisy	26	Caribbean/Canadian	College Diploma	Self-employed	Multigravida (4th Pregnancy)	Second
8	(PW31) Emma	31	Indian/ Canadian	Postgraduate	Full-time Employment	Primigravida	Third
9	(PW32) Francesca	25	Fijian/ Canadian	High School	Stay-at-home mom	Multigravida (2nd pregnancy)	Third
10	(OPW33) Gayle	Unspecified	Canadian	Postgraduate	Full-time Employment	Primigravida	Third
11	Heidi (PW34)	27	African	University Education	Full-time Employment	Primigravida	Third
12	Isabel (OPW35)	31	Asian	University Education	Part-time Employment	Primigravida	Second
13	Jessica (PW36)	32	Caucasian	University Education	Full-time Employment	Primigravida	Third
14	Kailani (PW37)	30	Caucasian	High School	Part-time Employment	Multigravida (2nd pregnancy)	Second

**Fig. 1:**
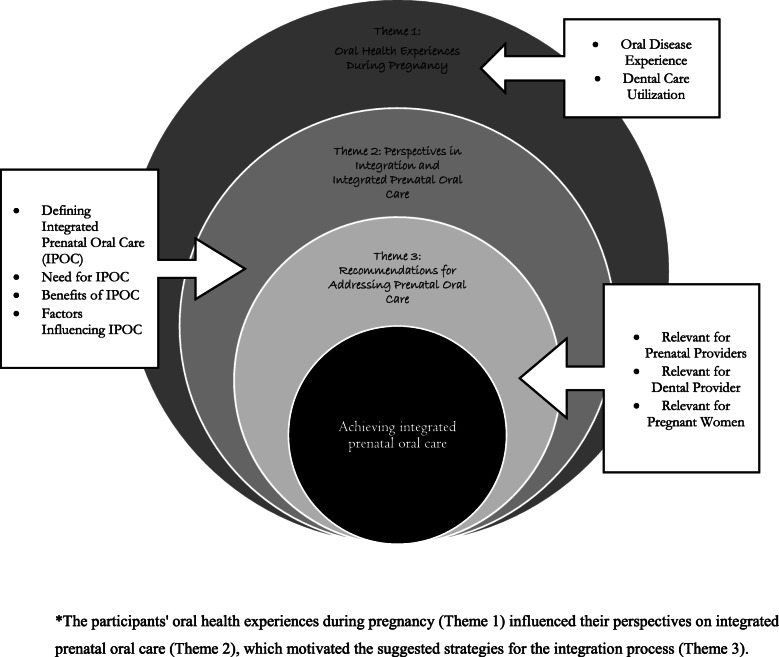
Thematic map of the identified themes that emerged from interview data*. *The participants’ oral health experiences during pregnancy (Theme 1) influenced their perspectives on integrated prenatal oral care (Theme 2), which motivated the suggested strategies for the integration process (Theme 3)

The interview lengths ranged between 28- and 65-min, the median time was 35 min. Three major themes depicted in Fig. 1 were elicited from the thematic analysis: (1) oral health experiences during pregnancy, (2) perspectives on integration and integrated prenatal oral care, and (3) strategies for addressing prenatal oral health care. Where possible, we used the participants’ own words under their pseudonyms to add veracity to their accounts [17].

### Theme 1: Oral health experiences during pregnancy

This theme was comprised of two subthemes: oral disease experience and dental care utilization. Regarding oral disease experience during pregnancy, eight of the participants reported having bleeding gums, sore gums and/or toothache. Seven participants reported doing nothing about the observed problems, although two mentioned discussing it with their prenatal providers: “*my mouth was bleeding all the time when I brushed my teeth. The doctor said it’s normal and pregnancy gingivitis was common… they didn’t really give me any solution*” (Wendy, second pregnancy). Most participants also noted that oral health was never addressed during prenatal care, including during the health education sessions.

In terms of dental care utilization, most interviewees recounted not seeking regular care from a dental professional during their current pregnancy “*even when I feel this pain, I kind of feel reluctant to go to the dentist*” (Anna, third pregnancy). Dental care was sought mostly for emergency situations, including for severe toothache, and a pregnancy tumor. Only two participants visited their dentists for routine dental care; one sought care based on advice from her prenatal provider. Two other participants mentioned having their routine dental visit cancelled because of the COVID-19 pandemic. Reasons for avoiding regular dental visits included fear (particularly of the safety of dental procedures during pregnancy), lack of insurance coverage and being unaware of the need for oral health care during pregnancy. In highlighting the importance of oral health awareness, one participant told us “*I have come from Pakistan where nobody knew about it [oral health] and nobody gets it checked. But then I came here nobody asked me to go to see a dentist or that you need to get it checked*” (Chloe, third pregnancy). This same participant had a severe toothache during her current pregnancy and stated that her negative experience could have been averted if she were better informed.

We also heard that “*dental visits are an additional cost, most people are not willing to shell out that money if they are not covered [insurance]*” (Emma, first pregnancy) and that “*my last pregnancy I had to borrow money to get my tooth extracted*.” (Daisy, fourth pregnancy). The general view was that “*dentists are expensive, and then when you get your teeth cleaned, that brings up other problems and like a never-ending pit of things that are wrong”* (Kailani, fourth pregnancy).

### Theme 2: perspectives on integration and integrated prenatal oral care

We identified four subthemes: defining integrated care, the need for integrated prenatal oral care, perceived benefits of this integration, and factors influencing delivery of integrated prenatal oral care. Participants seemed to define integrated care as “*multiple health care providers from different streams working together to ensure the patient’s good health*” (Emma, first pregnancy). Another participant explained what integrated care was not: “*referral is not integrated care in my book… [there’s] very little compliance*” (Gayle, first Pregnancy). This participant posited that a team approach is essential for integrated care. However, for most of the other participants, referrals were deemed critical, as is currently done with other procedures stating that “*they (referrals) are really important… they’re the main instrument for the integration*” (Zuriel, third pregnancy).

Most of the participants voiced a need for the inclusion of preventive oral health care in prenatal care. Some considered integration a superior approach because “*health care workers may not have all the answers, if another professional is able to help that would be helpful*” (Wendy, second pregnancy). In particular, we heard the idea of collocating dental professionals as part of the prenatal team “*to have it in one place [the prenatal team] would be like an ideal*” (Wendy, second pregnancy) in the prenatal team. Two participants referred to a coordinated system whereby care coordinators are engaged to support and make appointments for pregnant women. However, some participants did recognize that the physical presence of a dentist within the prenatal health care setting might not be feasible. In this case, they highlighted interprofessional education as an important factor in achieving integration, stating that “*fostering an integrated approach to learning right from medical school may be useful*” (Emma, first pregnancy). Interestingly, some participants stated integrated care was not required during pregnancy because “… *it’s not worth doing it for a pregnancy that’s going to last nine months”* (Gayle, first, pregnancy).

While considering the benefits, many participants viewed an integrated approach to oral health care as “*efficient in terms of time and resources*” (Anna, third pregnancy). Other benefits mentioned include better health and oral health status for the pregnant woman, fewer oral health-related complications, increased oral health awareness, early diagnosis and treatment, increased trust in dental providers, equity in care delivery, and reduced stress for the pregnant woman. One participant identified the potential impact of integration on self-worth, and mental well-being stating that they “*think they [pregnant women] would feel special, like valued… that would be good for self-confidence self-esteem*.” (Zuriel, third pregnancy). This was consistent with comments by another participant highlighting the fact that mothers often focus on their children, and may neglect themselves, “*I work 10 plus hours a day. When the day ends, it’s all about my kids*” (Daisy, fourth pregnancy). A few women mentioned the potential impact on the oral health of future generations: “*the most important part is once mom is aware of oral health that becomes important in the baby… so that they can have healthy teeth set up for a good start to their lives*” (Isabel, first pregnancy). They also commented about the timing: “*if they would tell me in the start then maybe I would be more enthusiastic to go*” (Chloe, third pregnancy).

The participants also identified a variety of factors that could facilitate the delivery of integrated prenatal oral care. The most commonly identified facilitator was the establishment of a simple process for addressing oral health during pregnancy “*they have to have a set system in place*” (Daisy, fourth pregnancy). The use of policies or guidelines regarding oral health for pregnant women was also mentioned as an important facilitator of inetgration, “*the only way it would happen would be through laws and policies*” (Zuriel, third pregnancy). Other facilitators identified included research evidence, advocacy, and establishment of communication channels between oral health and prenatal providers, and government support.

The participants mentioned a lack of political will, and the prevailing separate structure for care delivery as factors that could hinder integration. According to one participant, “*it appears dental care is considered a separate entity… the doctor addresses your body, and the dentist will address your head and neck area*” (Emma, first pregnancy). The majority of the participants identified the limited understanding of and value for oral health among non-dental health professionals and the public as a potential barrier to integration. In the words of one participant, “*I think first there is this view of dental care as secondary. It’s not a priority…. It’s seen as like a luxury thing… It’s not as important as a medical doctor*.” (Zuriel, third pregnancy). They also mentioned funding as a hinderance since “*some of those things are not affordable and especially if you’re a stay-at-home mom. You’re not covered [insurance]…*” (Francesca, second pregnancy). Apathy to the idea of integrated oral health care among health providers was also mentioned: “*changing anything in the health care system is difficult*” (Jessica, first pregnancy), and “*it’s gonna take way too much time on the doctors’ side with very little interest on the dentists’ side*” (Gayle, first pregnancy).

### Theme 3: strategies for addressing prenatal oral health care

Although a few participants posited that oral health care should be addressed even before pregnancy, the majority agreed that oral health “*should also be included as part of prenatal checks*” (Anna, third pregnancy), different perspectives were offered on how to proceed, including recommendations relevant to pregnant women, prenatal providers, and dental providers.

In terms of relevant recommendations for the pregnant women, participants identified oral health education and funding to enable integration. Oral health education was considered vital, with one participant stating they “*don’t think there’s like a lot of information about it out there. Not the way we talk about other things that have to do with prenatal care*” (Heidi, first pregnancy). Participants supported including oral health information on pregnancy apps and other online resources, and on leaflets that could be included in the prenatal package. In addressing funding issues, many participants supported the provision of free or subsidized dental care for women during pregnancy. The most common suggestion was for preventive oral health initiatives to fall under the Medical Services Plan (MSP): “*it would be pretty cool if it could be part of MSP, so we did not need to worry about the cost*” (Wendy, second pregnancy). One participant also suggested the use of vouchers for those who cannot afford dental care “*so that the women who need it can choose whichever dentist is closer to them*” (Daisy, fourth pregnancy).

For the strategies relevant to prenatal providers, participants highlighted the role of interprofessional education. Indeed, the prenatal providers were identified as critical in the integration process because “*those are the people that you’re going to have the closest relationship with…*” (Francesca, second, pregnancy), suggesting that “*having your prenatal provider encouraging people to go to the dentist would be really beneficial*” (Xena, first pregnancy) when a team approach is not present. Participants further recommended that prenatal providers should offer resources on oral health to all pregnant women. They proposed the use of oral health screening questions during the initial registration process, stating that “*the prenatal care provider should probe if somebody would need to go to the dentist or not*” (Francesca, first pregnancy). Although interprofessional education was suggested, there were mixed reactions to prenatal providers conducting oral examinations. Approximately half of the participants thought it would be acceptable, while the others preferred that trained dental providers conduct oral examinations: “*because they’re not oral health professionals. I don’t think that’s their task*” (Zuriel, third pregnancy). One participant also specified that oral examinations by non-dental providers may be undesirable to women with previous negative experiences such as a history of trauma.

For strategies relevant to dental providers, whether or not they are within the prenatal health care team, many participants suggested a dental provider should be visited during pregnancy with some stating that “*I just feel there should be one compulsory visit to a dentist*” (Anna, third pregnancy) in order to “*check teeth, gums, to see if there are small issues that could be resolved*” (Zuriel, third pregnancy). The participants proposed that dental providers should perform basic dental care, including dental checks, and cleaning for all pregnant women, while other non-urgent procedures should be delayed until after pregnancy. Many participants also suggested that dental providers should be advocates for the delivery of care to pregnant women.

## Discussion

We found that a majority of our participants supported including preventive and educational oral health care into routine prenatal care services via a variety of strategies. They considered the adoption of collaboration between prenatal and dental providers as imperative for successful integration. Figure 1 provides a summary of our understanding of the connections between the themes identified in the study. The participants’ oral health experiences during pregnancy (Theme 1) influenced their perspectives on integrated prenatal oral care (Theme 2), which motivated the suggested strategies for the integration process (Theme 3). This thematic figure is intended to show how the proposed strategies were derived. The schema is, however, limited by the fact that it may not reflect the perspectives of other stakeholders such as health care providers and policymakers. Interestingly, many of the items highlighted in the thematic map, mirror the dimensions highlighted in the rainbow model of integrated care as being necessary for effective integration [19].

In terms of their oral health experiences during pregnancy, our study participants reported not seeking dental care or discussing it with their prenatal provider even after developing dental symptoms similar to what was reported previously [9]. This finding underscores the low utilization of dental services reported among pregnant women in Canada [7–9]. The reasons proffered by the participants for non-utilization were also consistent with existing research evidence, these included low or no interest in oral health [11, 12, 20], lack of financial resources [7–9, 11, 12], and fear or concern about dental care during pregnancy [8, 11, 12, 20]. Such reasons demonstrate the need for oral health promoting activities integrated with prenatal care, given that only two participants received a referral to address the oral health complaints. Research evidence supports prenatal care providers as important for recommending dental care to pregnant women and making referrals [10].

Most of our participants considered an integrated care approach as involving multiple health providers working together for the patients benefit; they also shared their understanding that such care should be patient centred [14]. They identified good communication, collaborative practices, and a clearly outlined process as important factors for integrated care, which are all well recognized building blocks for successful integration [14, 21]. However, there was a lack of consensus regarding the most suitable type of integration for integrated prenatal oral care, with some participants recommending more than one type. Many suggested the collocation of services, while others suggested a coordinated approach; the least recommended approach was including an oral health provider in the prenatal team. This variation corroborates the current lack of a gold standard [16]. However, some interviewees viewed a referral system between oral health and prenatal providers as central to achieving integration irrespective of the integration type adopted [15].

Given the participants interest in referrals, and the fact that British Colombian medical and dental systems are financed differently and at present operate independently [22], linkages appears to be a feasible integration method— albeit, as a low level integration approach [15] as noted by some participants. Not surprising, some participants did not view referrals as integrated care. However, as acknowledged by most participants, referrals are currently employed for interaction between health care providers during prenatal care, and might therefore be easier to implement. Referrals could increase awareness of the need for oral health care during pregnancy and increase uptake of dental services during pregnancy [23] especially where interprofessional communication is adequate [10]. Some participants also supported using a care coordinator, as suggested in a previous study [23]. However, in line with the findings of an earlier study in Canada that prenatal care is complex and referral pathways between multiple health providers are not always clear [24], we posit that clearly outlined processes may be essential for successful integration of preventive oral health in prenatal care services.

The participants consider prenatal providers as playing a central role in the process, a view supported by Biordi and associates [25], who proposed a model using non-dental providers to increase access to oral health care. The participants specifically suggested including oral health assessments on the prenatal care procedure checklist. From their perspectives, the oral health assessments could start via simple questions during the initial prenatal visits, followed by a referral to a dental care provider. They also advocated that prenatal providers offer some basic oral health education during prenatal care, which requires interprofessional education. However, they expressed concerns about the capacity of prenatal providers to conduct oral examinations. While some women supported the administration of brief oral examinations by prenatal providers, others posited that only oral health providers should conduct oral examinations— even if simple and basic. Such views go against research reports providing evidence that with proper training non-dental providers are capable of conducting brief oral examinations [25–27].

The suggestion by some participants that interprofessional education may be critical for achieving integrated care is important since there is currently minimal collaboration between dental and non-dental providers [28]. Thompson et al. ([29] also recommended educating prenatal providers about pregnancy-specific oral care so they can serve as oral health advocates. Interprofessional education will likely foster the development of collaborative practices between dental and non-dental providers. Research evidence supports the need for and value of, interprofessional education for both dental and non-dental providers in achieving integrated care [30]. Another study [27] confirmed the positive impact of pre-doctoral oral health training on medical students’ behaviors, confidence, and attitudes related to prenatal oral health care. In addition, a previous study [31] confirmed that interprofessional education increased access to care for children in their study.

All the participants recognized funding as a major barrier to oral health care access during pregnancy. They recommended including oral health in the MSP or the use of vouchers for women who cannot afford care; they were in support of publicly funded dental care which has been advocated at length for Canadians [32]. Similar strategies have been reported in the literature as useful for addressing funding barriers for oral health care. Therefore, we suggest the development of funding programs to address the oral health needs of pregnant women, especially those with limited access to oral health care. As mentioned by a participant, it may also serve as a boost for their mental well-being and quality of life [6]. Using vouchers is also a viable option [33], as priority could be given to socially disadvantaged women who experience additional oral diseases and have less access to oral health care [7–9]. Traditionally, however, medical and dental services operate independently in Canada; these two services are also funded differently. Nonetheless, there has been support for adopting an integrated approach for the concurrent delivery of these two health care services together [16], particularly for those who have access to medical but not oral health care services [26, 34]. The progress towards achieving this integration has been slow, however [21].

Concerns regarding the provision of dental care during pregnancy, which was also identified by others [7, 8], need to be discussed further. While increased oral health awareness may be useful in reducing concerns, the delivery of culturally sensitive and trauma-informed care must be implemented. In fact, the inclusion of cultural sensitivity in the training curriculum of health care providers has been recommended [31] as a strategy for fostering a perception change on seeking dental care. Similarly, a trauma informed approach may help to create a safe environment. Notably, a few participants mentioned that integrated care was not required during pregnancy. Such discordant beliefs might be related to a preconception that dental care is not necessary during pregnancy, which corroborates with other studies [8, 20]. The preconceptions of an individual may possibly be related to action; however, we did not explore the reasoning behind this belief.

The strengths of this study include exploring the views of a purposefully selected sample of diverse women from across different settings, achieving data saturation, and using strategies to ensure rigor in the data analysis process (such as member checking, field notes, and an audit trail). However, the study has limitations, such as the inclusion of women who only spoke English, which excluded the views of non-English-speaking women who may share different cultural values and beliefs. Also, participants were drawn from two large cities in BC, and the perspectives of women residing in other cities, or from remote and rural areas were absent. Furthermore, qualitative studies, as such, do not intend to generate generalizability but to provide deeper understanding of the phenomenon. As may be expected from an interview-based study, social desirability might have also affected participants’ responses. As an oral health professional who has experienced pregnancy and has a strong interest in prenatal the oral health care; the interviewer’s experiences may have influenced the interviews or interpretation of the study findings. Using field notes, memoing, member checking, and having multiple researchers conducting the coding and thematic analysis may address this potential bias. Lastly, we did not explore the extent to which the current pandemic affected participants’ views on the issues of concern Nevertheless, our study findings provide an overview of how pregnant women would like to have oral health addressed during prenatal care. We suggest further studies among a larger and more diverse group of pregnant women to ensure generalizability of the study findings.

## Conclusion

Pregnant women in British Columbia support the inclusion of educational and preventive oral care during prenatal care visits, although their views differed in terms of how such inclusion can be achieved. They also advocated including oral assessments in the prenatal checklist and establishing a referral system as acceptable strategies for providing integrated prenatal oral health care. Finances remain a major barrier for accessing oral health care.

## Supplementary Information

**Additional file 1.** Integrating oral health in porenatal care in-depth interview guide.

## Data Availability

The datasets used and analysed during the current study are available from the corresponding author on reasonable request.

## Abbreviations

BC: British Columbia
COVID-19: Coronavirus disease 2019
MSP: Medical services plan
WHO: World health Organization
