# Supplementary material for: Pregnant women’s perspectives on integrating preventive oral health in prenatal care
Source: BMC Pregnancy Childbirth. 2021 Apr 1;21:271. doi: 10.1186/s12884-021-03750-4 (PMC8016156; doi:10.1186/s12884-021-03750-4)
Supplement: Supplementary file 1 — Additional file 1. Integrating oral health in porenatal care in-depth interview guide. [file 12884_2021_3750_MOESM1_ESM.pdf]

In-Depth Interview Guide

Pregnant women's perspectives on integrating preventive oral health in prenatal care.

Adeniyi A*1, Donnelly L2, Janssen P3, Jevitt C4, Kardeh B5, von Bergmann H6, Brondani M7

<u>Introduction and Ground Rules (5 minutes)</u>

Good day and thank you for making out time for this interview. My name is Abiola Adeniyi and I will be seeking your views on the inclusion of preventive oral health services in routine prenatal care.

<u>Purpose</u>: I am specifically interested in learning about your experiences with the delivery of preventive oral healthcare services during routine prenatal care and your views on whether and how preventive oral healthcare services should be included in prenatal care. I expect the interview to last no more than sixty minutes.

<u>Confidentiality</u>: Everything discussed today will remain confidential and will be tape-recorded. I may put some of your comments in a summary of this discussion but no names or other identifying information will be used anywhere. Your participation is voluntary and you can decide to end the interview at any point, or to refrain from answering any question you do not feel comfortable with.

<u>Note taking and recording</u>: I will be taking notes and recording the discussion so that we can have a true account of what was said and so we don't miss anything. Your comments will be anonymous in the transcribed version and the audio version will be destroyed

Is there anything you will like to ask me before we start the interview?

Thank you.

Socio-demographic information

Before we start today I would appreciate it if you could answer a few questions about yourself.

1. Can you tell me a little about yourself?
   - Year of birth
   - Gender
   - Profession
   - Ethnicity
   - Work experience
   - Number of previous pregnancies

Introduction

Please read the vignette below, following which I would like to ask a few questions on the scenario. Please read the scenario below. After you read the scenario, I would like to ask you a few questions about it.

Tia is a 32-year-old woman. She is 6 months pregnant with her first child. She moved into the neighbourhood two weeks ago and she is single. She is visiting the local maternity clinic today to enroll for routine prenatal care.

Before moving to this location her prenatal care was provided by a doctor. Her first doctor gave her a detailed letter of referral. According to her former doctor, her pregnancy is progressing well with no problems. During the initial enrolment procedures at the clinic, Tia mentions that she smoked 5 cigarettes daily for about 10 years. She stopped when she got pregnant.

She also mentioned that she had a sharp pain in a tooth in her lower jaw a week ago. Her gums bleed when she brushes her teeth. The bleeding started during the pregnancy. On further questioning, Tia

mentioned that before getting pregnant food was getting into a hole in one tooth. That tooth is around where she felt the pain last week. Tia did not visit a dentist for care because she has no dental insurance. She could not afford to pay the dentist's fees. She is afraid to go to the dentist because she is not sure how dental treatments would affect her baby. Tia still does not have dental insurance coverage. Her last visit to the dentist was about 2 years ago. Otherwise Tia is fine and her pregnancy is progressing well.

2. In your experience, how common is this scenario among pregnant women? Do you think her experience of pain could have been prevented? Please explain your answer.
3. Can you tell me a little about your experience with oral health and oral health services during pregnancy?
4. Can you tell me what you think should be done about Tia's dental complaint?
   - Based on your experience can you describe how dental complaints/problems like Tia's are handled during pregnancy.
   - Do you think you could help her in. any way and how? *(Administrators and HCP)*
5. Can you tell me why you think Tia did not seek dental care?
   - What factors not mentioned in the scenario do you think may explain why she did not seek care?
6. Can you describe what could happen if Tia does not seek dental care?
   - Any effect on her on her unborn child?
7. How do you think her experience of pain could have been prevented? Please explain your answer.

Views on access to preventive oral health during prenatal care in BC

8. In your view what would influence Tia's ability to access preventive oral care?
9. Do you think Tia would have benefitted from any policy/guidelines addressing preventive oral care in during pregnancy? *(Examples of policy - Seatbelt policy/ smoking in public places/ helmet)*
   - Are you aware of any such policy/guidelines?
   - Could you describe how these guidelines for oral healthcare in pregnancy been implemented generally in BC? What about the implementation in your facility?
   - If you were a policymaker what would you recommend to address oral health during pregnancy?
10. How is preventive oral healthcare for the pregnant woman currently being addressed during prenatal care in your healthcare facility?
    - Is preventive oral healthcare offered to pregnant women in your facility?
    - Could you describe what dental services are offered, how they are offered and by whom?
    - Why do you think oral health is addressed in this way?
    - Why do you think a dentist or a dental hygienist would be necessary?

Views on providing preventive oral health in prenatal care

11. Can you tell me your views on how preventive oral healthcare can be addressed during pregnancy?
12. Can you tell me what you think prenatal health workers can do about preventive oral healthcare during pregnancy? What about oral healthcare workers?
13. What are your thoughts on including preventive oral healthcare in routine prenatal care? Please explain.
    - What sort of preventive oral healthcare services do you think can be provided during pregnancy?
    - How do you think the pregnant women like Tia will feel about receiving these services as part of routine antenatal care?
    - How do you think prenatal care workers would feel about including preventive oral health in prenatal care?
    - How do you think oral health workers would feel about including preventive oral health in prenatal care?
    - Do you think it is necessary for oral healthcare providers and prenatal providers to collaborate to provide preventive oral healthcare for pregnant women like Tia? How do you think this should happen and why?
    - Do you think including preventive oral health in prenatal care is an achievable goal? Please explain.
    - If you have to include preventive oral healthcare during pregnancy how will you go about this?

Models for integrating oral health in prenatal care

14. Considering what we just discussed, there are models of how things should happen. I would like to show you an example of such a model. What do you think of this model (*present the oral health integration model*) for including oral health in prenatal care? How do you see this model working for you? Would you utilise this model?
    - Would you suggest any change to the model? Please explain
15. What do you think of the proposed model for providing care in this model?
    - Would you suggest any change to the model? Please explain

Barriers and facilitators

16. What in your opinion would make the inclusion of oral health in prenatal care easy to achieve?
17. What specific challenges would you associate with including preventive oral healthcare such as oral health assessments, oral health promotion and referrals for dental care into care of women attending antenatal clinics?
    - Could you suggest strategies for addressing these challenges
18. Is there anything that you might not have thought about before that you think may be relevant for this interview?
19. Is there anything else you think I should know to understand better?

If there any questions I would be happy to answer them.

Model of Integrated Oral healthcare

Adapted from WHO 2016 [56], and Curry & Ham [71].

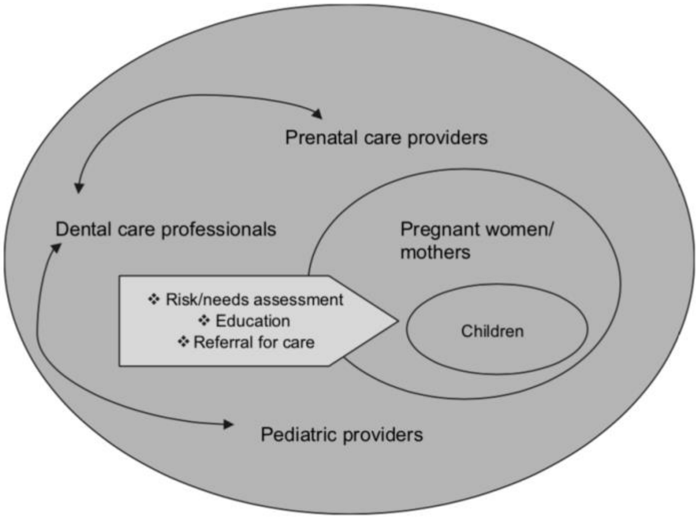

FIGURE 1
Oral health integration model.

This model has been used for integrating oral healthcare into both prenatal care and pediatric care. The model recommends that prenatal and pediatric care providers screen ALL patients for oral diseases using the oral health delivery framework and then refer those needing care to the dentist.
